# Akt Activation Correlates with Snail Expression and Potentially Determines the Recurrence of Prostate Cancer in Patients at Stage T2 after a Radical Prostatectomy

**DOI:** 10.3390/ijms17081194

**Published:** 2016-07-23

**Authors:** Wei-Yu Chen, Kuo-Tai Hua, Wei-Jiunn Lee, Yung-Wei Lin, Yen-Nien Liu, Chi-Long Chen, Yu-Ching Wen, Ming-Hsien Chien

**Affiliations:** 1 Graduate Institute of Clinical Medicine, College of Medicine, Taipei Medical University, Taipei 110, Taiwan; 1047@tmu.edu.tw; 2 Department of Pathology, School of Medicine, College of Medicine, Taipei Medical University, Taipei 110, Taiwan; 3 Department of Pathology, Wan Fang Hospital, Taipei Medical University, Taipei 116, Taiwan; 4 Graduate Institute of Toxicology, College of Medicine, National Taiwan University, Taipei 100, Taiwan; d94447003@gmail.com; 5 Department of Medical Education and Research, Wan Fang Hospital, Taipei Medical University, Taipei 116, Taiwan; lwj5905@gmail.com; 6 Department of Urology, School of Medicine, College of Medicine, Taipei Medical University, Taipei 110, Taiwan; 7 Department of Urology, Wan Fang Hospital, Taipei Medical University, Taipei 116, Taiwan; highwei168@gmail.com; 8 Graduate Institute of Cancer Biology and Drug Discovery, College of Medical Science and Technology, Taipei Medical University, Taipei 110, Taiwan; liuy@tmu.edu.tw

**Keywords:** prostate cancer, radical prostatectomy, stage T2, Akt, Snail, biochemical recurrence

## Abstract

Our previous work demonstrated the epithelial-mesenchymal transition factor, Snail, is a potential marker for predicting the recurrence of localized prostate cancer (PCa). Akt activation is important for Snail stabilization and transcription in PCa. The purpose of this study was to retrospectively investigate the relationship between the phosphorylated level of Akt (p-Akt) in radical prostatectomy (RP) specimens and cancer biochemical recurrence (BCR). Using a tissue microarray and immunohistochemistry, the expression of p-Akt was measured in benign and neoplastic tissues from RP specimens in 53 patients whose cancer was pathologically defined as T2 without positive margins. Herein, we observed that the p-Akt level was higher in PCa than in benign tissues and was significantly associated with the Snail level. A high p-Akt image score (≥8) was significantly correlated with a higher histological Gleason sum, Snail image score, and preoperative prostate-specific antigen (PSA) value. Moreover, the high p-Akt image score and Gleason score sum (≥7) showed similar discriminatory abilities for BCR according to a receiver-operator characteristic curve analysis and were correlated with worse recurrence-free survival according to a log-rank test (*p* < 0.05). To further determine whether a high p-Akt image score could predict the risk of BCR, a Cox proportional hazard model showed that only a high p-Akt image score (hazard ratio (HR): 3.12, *p* = 0.05) and a high Gleason score sum (≥7) (HR: 1.18, *p* = 0.05) but not a high preoperative PSA value (HR: 0.62, *p* = 0.57) were significantly associated with a higher risk of developing BCR. Our data indicate that, for localized PCa patients after an RP, p-Akt can serve as a potential prognostic marker that improves predictions of BCR-free survival.

## 1. Introduction

Globally, prostate cancer (PCa) accounts for 15% of male cancers and 6.6% of total male cancer mortality [1]. A radical prostatectomy (RP) is recognized as the gold standard for treating patients with localized PCa. The most important advantage of an RP is its potential to cure without damaging adjacent tissues and provide accurate staging because of the total removal of the organ. Although most patients are cured after surgery, around 23%–35% of PCa patients progress to biochemical recurrence (BCR) due to serum prostate-specific antigen (PSA) elevation, indicating that they have an increased risk of developing advanced PCa among 10 years after an RP [2,3]. To now, the challenge of PCa patients after an RP has been to determine which patients harbor high-risk disease requiring aggressive/curative therapy and which patients harbor indolent disease that can be managed with active surveillance.

Clinical prognostic risk factors such as the Gleason score, pathological stage, a positive surgical margin, and preoperative PSA value are used to estimate patient outcomes postoperatively [4,5]. However, the sensitivity of predicting BCR of individual patients using such parameters is insufficient [4,5]. Hence, novel biomarkers are needed to predict BCR in PCa patients after an RP to help provide better patient counseling, to help with more-precise clinical decision-making, and to search for therapeutic targets. Recently, studies have identified several molecular alterations involved in prostate recurrence. For example, we previously identified that the epithelial-mesenchymal transition (EMT) factor, Snail, is upregulated in PCa and is a predictive factor for subsequent localized PCa recurrence after an RP [6]. However, the precise mechanisms underlying Snail expression in this malignancy has not been fully elucidated.

Activation of the serine threonine kinase, Akt (phosphorylated (p)-Akt), was reported to regulate the stability and transcription of Snail in several cancer types, such as colorectal [7], oral [8], and prostate [9] cancers. A previous report indicated that p-Akt was expressed in around 8% of non-neoplastic prostate and 50% of PCa cases, indicative of its overexpression in cancer [10]. Increased Akt phosphorylation was observed in high-Gleason-score PCa and was correlated with proliferation in human PCa as estimated by the expression of the cell proliferation antigen, Ki67 [11,12]. Bedolla et al. recruited 65 PCa patients including T1~T3 stages with positive margins and showed that p-Akt is an important predictor of the risk of BCR [13]. Based on these results, we hypothesized an important role for Akt in PCa recurrence.

To further investigate the role of Akt activation in localized PCa recurrence, this study recruited 53 PCa patients at the T2 stage without positive margins after an RP. We evaluated the p-Akt expression pattern in these PCa patients using immunohistochemistry (IHC), and correlated expression levels with Snail and other clinicopathological parameters. We report for the first time that expression of p-Akt was highly correlated with Snail expression in localized PCa, and the cytoplasmic p-Akt protein level has potential to serve as an independent biomarker to improve estimation of localized PCa prognoses.

## 2. Results

In this study, we recruited 76 PCa patients who had not received neoadjuvant therapy and had undergone a whole-mount pathological assessment of their tumor after an RP. Next, we further excluded patients with a positive surgical margin and seminal vesicle invasion, and 53 of 76 patients who had organ-confined disease were ultimately recruited. Demographic and clinical characteristics are summarized in Table 1. Among the 53 PCa patients, the age at the time of the RP ranged 48–88 (mean, 70.7 ± 15.2) years. The histologic type of all tumors was an adenocarcinoma. According to the American Joint Committee on Cancer (AJCC) TNM staging system, tumors were classified into T2a (*n* = 6), T2b (*n* = 4), and T2c (*n* = 43). At a mean follow-up time of 71 months, 25 of 53 patients had BCR.

**Figure 1:**
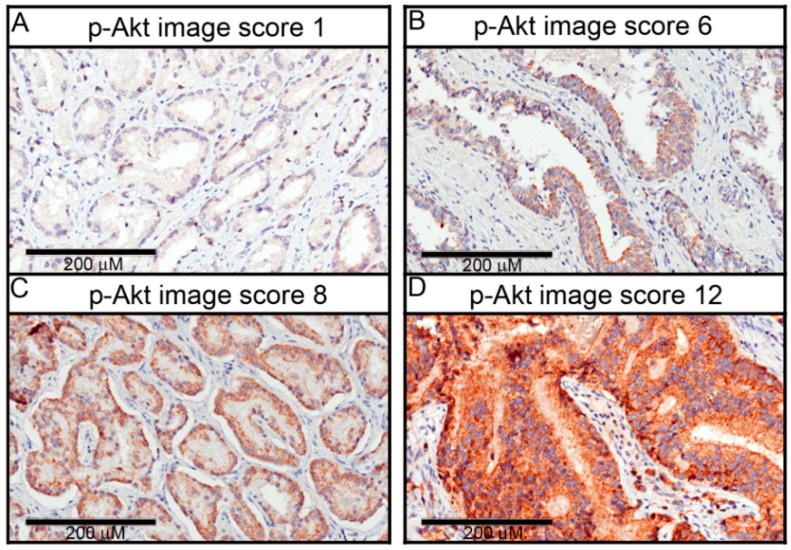

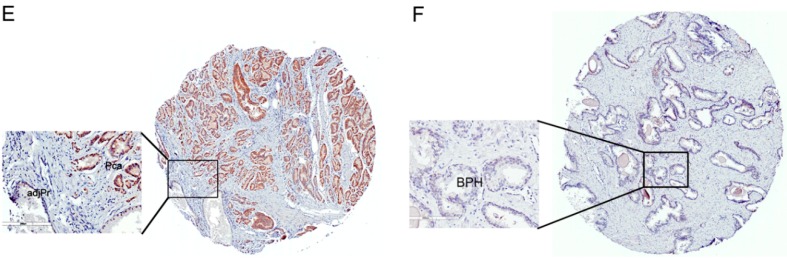
Phosphorylated (p)-Akt expression levels in representative primary prostate cancer (PCa) and non-neoplastic prostate tissues. Tissue microarrays (TMAs) of primary PCa and non-neoplastic prostate (benign prostatic hyperplasia; BPH) tissues were immunohistochemistry (IHC) analyzed for p-Akt. (**A**) Patient with a weak p-Akt expression level (intensity score 1 × extent score 1 = p-Akt image score 1); (**B**) Patient with T2cN0M0 cancer, a Gleason score of 3 + 4 = 7, and a moderate p-Akt expression level (intensity score 2 × extent score 3 = p-Akt image score 6); (**C**) Patient with T2cN0M0 cancer, a Gleason score of 4 + 3 = 7, and marked p-Akt immunostaining in the cytoplasm (intensity score 2 × extent score 4 = Snail image score 8); (**D**) Patient with T2cN0M0 cancer and a Gleason score of 4 + 5 = 9 and who displayed marked p-Akt immunostaining in the cytoplasm and discrete, diffuse staining in the nucleus (intensity score 3 × extent score 4 = image score 12) (200×); (**E**,**F**) No p-Akt immunostaining signal was detected in non-tumor adjacent tissues (**E**) or BPH (**F**). The high-power fields (200×) are magnified fields in the black boxed area in the right panel.

Figure 1A–D shows that p-Akt expression was observed in PCa tissue with a wide distribution of IHC scores. Immunostaining was almost completely restricted to the cytoplasm of epithelial tumor cells, and the pattern of expression was usually homogeneous. The p-Akt score was determined by multiplying the staining intensity (1–3) by the distribution rate (1–4) to represent p-Akt expression in PCa tissues, and representative examples of tumors showing overall low (with an image score of ≤6) and high (with an image score of ≥8) p-Akt expressions are illustrated in Figure 1A–D. In contrast to PCa, non-tumor adjacent tissues or benign prostatic hyperplasia (BPH) expressed p-Akt very weakly or not at all (Figure 1D,E), indicating that high levels of p-Akt were almost exclusively expressed in cancer tissues.

**Figure 2:**
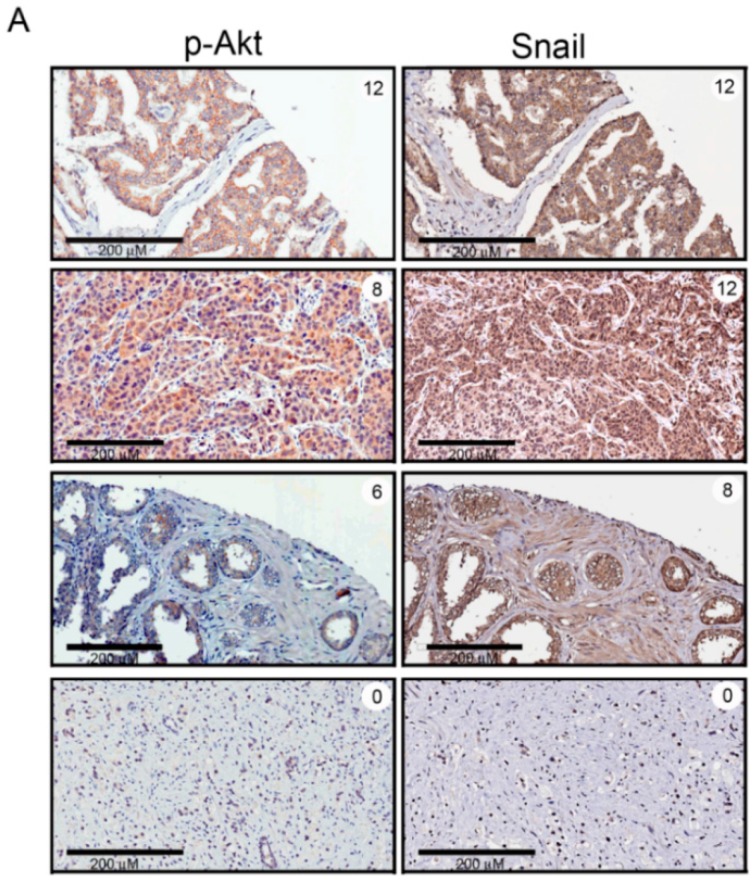

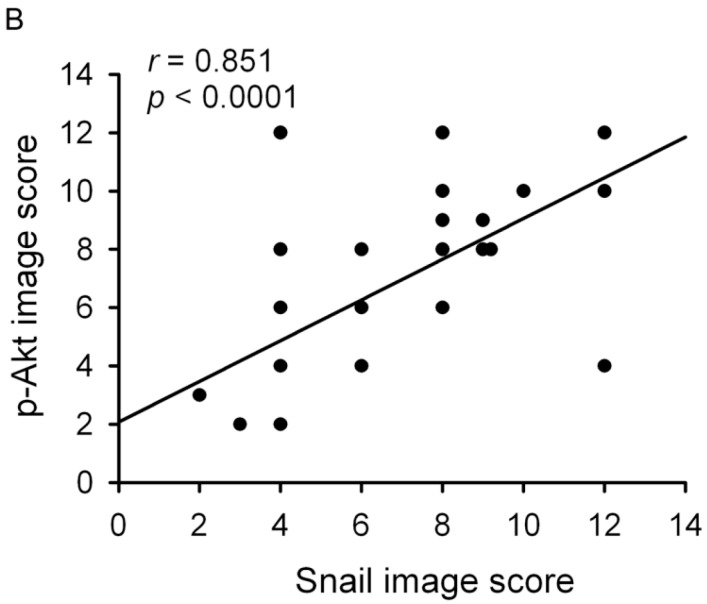
Phosphorylated (p)-Akt expression is positively correlated with Snail protein levels of patients with localized prostate cancer (PCa). (**A**) IHC staining analysis of p-Akt and Snail proteins in serial sections (200× magnification). Note the positive correlation of p-Akt and Snail protein expressions in tumor cells; (**B**) A significant positive correlation was observed between p-Akt expression levels and Snail expression levels (Spearman’s correlation coefficients: *r* = 0.851, *p* < 0.0001).

Previous studies indicated that Akt activation is important for Snail stabilization and transcription in PCa cells [9,14]. To further examine the correlation between expression levels of p-Akt and Snail in PCa, the same PCa TMA cohort was used. Representative IHC staining of p-Akt and Snail with different image scores on serial section of the same patients are shown in Figure 2A. IHC analysis of PCa specimens revealed a significant positive correlation between p-Akt and Snail expressions (Spearman correlation coefficient *r* = 0.851, *p* < 0.0001; Figure 2B).

**Figure 3:**
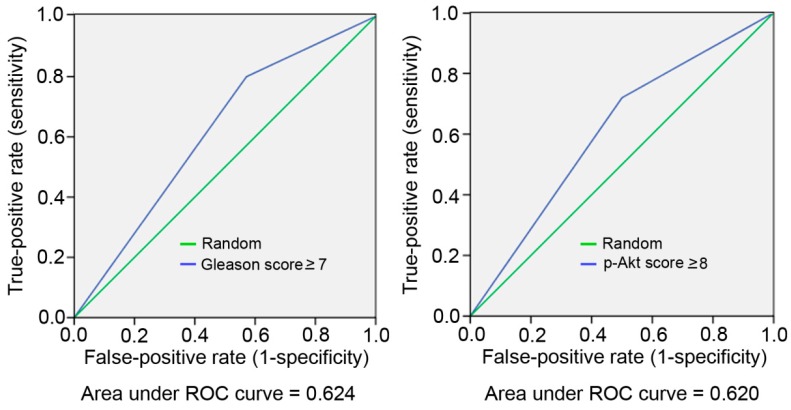
Sensitivity and specificity of gain of a high Gleason score or high p-Akt in specimens with respect to biochemical recurrence (BRC). Areas under the ROC (AUC) for high p-Akt image score (≥8) and high Gleason score (≥7) were 0.62 and 0.624, indicating similar discriminatory abilities for BRC.

As we showed earlier [6] , staining for Snail was significantly correlated with postoperative BCR of PCa. We further investigated relationships between p-Akt expression and selected clinicopathologic factors. Table 2 shows that among the 53 recruited patients, 32 patients (60.4%) were identified as having a high p-Akt image score (of ≥8), and the remaining 21 patients had a low p-Akt image score (of ≤6). High p-Akt (score of ≥8) expression was significantly associated with a higher histological Gleason sum (score of ≥7) (*p* = 0.024), Snail image score (score of ≥8) (*p* = 0.035), and preoperative PSA value (*p* = 0.026). Moreover, we also observed that high p-Akt expression was significantly correlated with postoperative BCR (*p* = 0.012). Moreover, according to an ROC analysis, the areas under the ROC curve for high p-Akt image score (score of ≥8) and Gleason score sum (score of ≥7) were similar, indicating that the high p-Akt image score and Gleason score sum showed similar discriminatory capacities for BCR (Figure 3).

**Figure 4:**
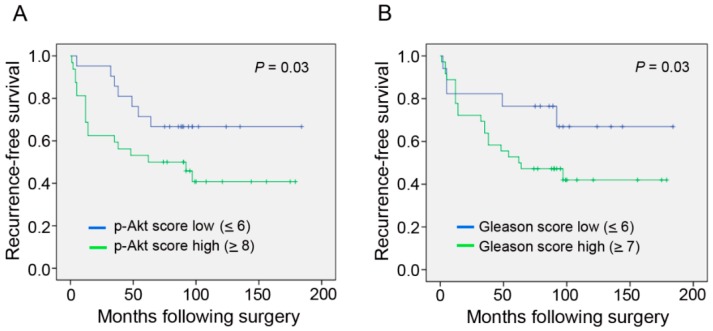

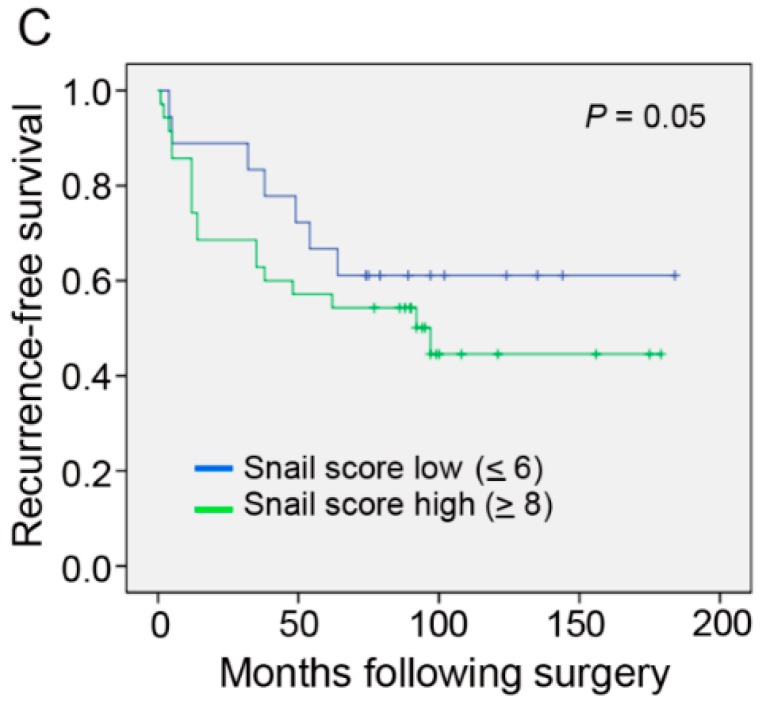
Kaplan-Meier survival curves showing relationships of the phosphorylated (p)-Akt image score (**A**), Gleason score sum (**B**), and Snail image score (**C**) in primary tumors with recurrence-free survival in 53 patients with clinically-localized prostate cancer. The recurrence-free survival of patients with a higher p-Akt, Snail image score (≥8) or Gleason score sum (≥7) was significantly lower than that of patients with a lower p-Akt, Snail image score (≤6) or Gleason score sum (≤6) (*p* ≤ 0.05, log-rank test).

According to the Kaplan-Meier test, we observed that patients with higher p-Akt expression (with scores of ≥8) had shorter recurrence-free survival times compared to those with lower expression (with scores of ≤6) of the protein (Figure 4A). For patients who had higher p-Akt tumor expression, the median recurrence-free survival was 62 months, whereas for those who demonstrated lower p-Akt tumor expression, it was 88 months (*p* = 0.03) (Figure 4A). Moreover, results of the Kaplan-Meier test also showed that patients with a higher Gleason score sum (of ≥7) or a higher Snail expression (with a score of ≥8) all had significantly shorter recurrence-free survival times (*p* = 0.03 and 0.05) (Figure 4B,C). These results showed that the *p* value of the Kaplan-Meier test used to compare the higher p-Akt group was the same and smaller than the higher Gleason score group and higher Snail group, respectively.

A Cox proportional hazard model was conducted to further explore relationships of p-Akt and Snail expressions with recurrence-free survival of the 53 patients with PCa after an RP. Table 3 summarizes the associations between the recurrence-free survival rate of the 53 patients with PCa and clinicopathologic parameters. In this analysis, we only observed that a high p-Akt image score (≥8) (hazard ratio (HR): 3.12, *p* = 0.05) or a high Gleason score sum (≥7) (HR: 1.18, *p* = 0.05), but not a high pre-operative PSA value (>10 ng/mL) (HR: 0.62, *p* = 0.57), was significantly correlated with worse recurrence-free survival (Table 3). In conclusion, our results suggest that a high p-Akt image score and a high histological Gleason score sum but not the preoperative PSA value can predict organ-confined PCa recurrence in our study. Moreover, we observed that patients with a high Snail image score (≥8) also tended to correlate with BCR (HR: 1.31, *p* = 0.06). Furthermore, our data indicated that patients with a high p-Akt image score (HR: 3.12) showed a higher risk for BRC than patients with a high Gleason score sum (HR: 1.18) or a high Snail image score (HR: 1.31) (Table 3).

## 3. Discussion

PCa is the most common cancer and the second leading cause of male cancer deaths in the United States [15]. This underscores the need for a more-thorough molecular understanding of this resilient disease. Generally, patients with clinically-localized PCa will be cured after receiving radical surgery. However, a fraction of patients with localized PCa harbor microscopic localized or metastatic residual disease. The lethal consequences of PCa are related to its metastasis to other organ sites. Although the preoperative PSA value, surgical margin status, and Gleason score sum have been extensively used in assessing biochemical disease recurrence risk after RP, the sensitivities of these approaches are insufficient [16,17]. Therefore, it is of critical significance to discover a new marker for the early prediction of tumor recurrence, and earlier adjuvant therapy is very important for clinicians.

The EMT is a critical cellular mechanism during tumor progression and development of metastasis. It was suggested that the EMT is co-opted by PCa cells during their metastatic dissemination from a primary organ to secondary sites [18]. We previously showed that increased expression of the EMT promoter, Snail, in the prostatic epithelium is a good predictor of BCR following a prostatectomy [6]. The phosphatidylinositol 3’-kinase (PI3K)/Akt pathway is frequently activated in various cancers and plays an important role in promoting the EMT through regulating Snail stability in PCa [9,19]. Our current results indicated that the Akt activation status was significantly correlated with Snail expression levels in tissues from patients with clinically-localized PCa (T2 stage only). Representative IHC staining patterns of p-Akt and Snail from consecutive serial sections were nearly identical in PCa specimens, further implying their highly correlated expressions. Compared to our previous study [6], we extended the postoperative follow-up time (an average of 51 to 71 months) of localized PCa patients and further investigated the correlation between the Akt activation status in PCa specimens and BRC of patients. Our data showed that p-Akt was predominantly expressed in PCa, but not in non-neoplastic tissues. Cox proportional hazard models suggested that the p-Akt index (HR: 3.12, *p* = 0.05) is a better postoperative marker than the preoperative PSA value (HR: 0.62, *p* = 0.57) in localized PCa in our patient cohort. Although the Gleason score sum showed a similar discriminatory capacity with the p-Akt index and was also a useful predictor of BRC in our patient cohort, it was not as good as p-Akt. Compared to a high p-Akt image score, a high Gleason score sum showed a lower HR (HR: 1.18, *p* = 0.05) for BRC. Moreover, our previous study [6] indicated that the Snail index might be a useful predictor of BRC in the same patient cohort. However, after we extended the postoperative follow-up time in this study, the high Snail image score only showed a borderline significant trend (*p* = 0.06) of correlating with BRC, suggesting that p-Akt might show higher sensitivity than Snail for predicting organ-confined PCa recurrence. In addition to Snail, other transcription factors such as Slug, Zeb1, and Zeb2 are also involved in the control of EMT [20]. A previous report indicated that Akt activation can upregulate Snail and Zeb2 and promote EMT in squamous cell carcinoma [21]. However, the roles of Akt and Zeb2 in prostate cancer progression and recurrence are still unclear and worth further investigation in our future work.

High levels of p-Akt are associated with earlier recurrence, clearly indicating that p-Akt is associated with aggressiveness and disease progression in PCa. In addition to the Akt-mediated Snail expression and EMT induction in an androgen-independent PC3 cell line [14], Akt was also shown to be involved in a number of proliferative, metabolic, and antiapoptotic pathways that are dependent on PI3K signaling for activation [22]. Activated Akt was suggested to regulate a number of intracellular targets such as p27^Kip1^, Bcl-2-associated death promoter (BAD), and caspase-9 which are involved in PCa progression and androgen independence [23,24,25]. Androgen deprivation therapy on androgen-dependent PCa cells such as LNCaP was reported to stimulate Akt activation, which finally resulted in androgen independence of the cells [26]. The pro-survival role of Akt activities was further shown in several clinical studies. For instance, increased levels of Akt or p-Akt expression were associated with a high Gleason grade and a worse prognosis in PCa [27,28,29]. Herein, we also showed that high levels of p-Akt were associated with more-aggressive features of the disease, as patients with high levels of p-Akt were identified as having a high PSA level and a high Gleason score. 

Although the clinical application of p-Akt in predicting BCR of PCa was previously reported [10,13], the patient cohort recruited in this study was totally different from those of previous studies. Previous studies [10,13] enrolled PCa patients after an RP at different stages (pT1–pT3), and included those with lymph node metastasis, extracapsular extension, positive margins, and seminal vesicle invasion. However, in our study, we excluded PCa patients with positive margins or seminal vesicle involvement and only included true organ-confined PCa patients (pT2a–pT2c) after an RP. To our best knowledge, this is the first report to investigate the relationships of p-Akt and Snail with the prognostic role of p-Akt in patients with clinically-localized PCa. The pathological definition of T2a–T2c stages is involvement of tumor cells in the prostate: in T2a, the tumor involves ≤50% of one lobe; in T2b, the tumor involves >50% of one lobe, but not both lobes; and in T2c, the tumor involves both lobes [30]. A previous report indicated that tumor focality can significantly influence the BCR-free survival rate [31]. However, more-recent reports indicated that tumor focality did not predict the risk of BCR after an RP in men with clinically-localized PCa, even if the tumor involved both lobes of the prostate [32], suggesting that tumor focality might not be a suitable predictive marker for BCR in patients with organ-confined PCa. Our study observed that p-Akt expression level should be a better predictive marker of BCR in this PCa population. In addition to Akt, its downstream signaling pathways such as GSK-3β inactivation and NF-κB activation were also involved in Akt-mediated Snail expression in prostate cancer cells as well as in other cancer types [7,14,21]. Our future work will further investigate the correlation between these downstream effectors and BCR-free survival rate in patients with localized PCa.

## 4. Materials and Methods

### 4.1. Patient Selection and Specimen Collection

Pathology files of Wan Fang Hospital and Taipei Medical University Hospital were searched, and 76 radical prostatectomy specimens with a pathologic diagnosis of prostatic adenocarcinoma were found from March 1999 to December 2011. The pathologic diagnosis and Gleason scoring were microscopically reconfirmed by pathologists. Each case was pathologically staged using the 2002 American Joint Committee on Cancer TNM staging system. In our recruited patients, 13 patients with advanced stage were excluded. Another 10 patients who had positive surgical margins were also excluded from the study. Ultimately, 53 cases fulfilling the selection criteria were included for further study. Follow-up information was obtained from a cancer registration database. A PSA level of ≥0.2 ng/mL on at least two occasions over a 2-month period was used to define biochemical failure [33].

### 4.2. Tissue Microarrays (TMAs)

Two independent PCa TMA sets were used in this study. PCa samples from patients were obtained with informed consent (Taipei Medical University-Wan Fang Hospital Institutional Review Board No. 99049). TMAs were constructed using a manual tissue-arraying instrument (Beecher Instruments, Sun Prairie, WI, USA). Briefly, carcinoma areas were manually punched, and duplicate tissue cores measuring 2.5 mm in diameter were inserted into recipient paraffin blocks. Sections measuring 5 μm in thickness were cut and transferred to glass slides. The presence of tumor tissue was further verified on a hematoxylin and eosin (H and E)-stained section.

### 4.3. Immunohistochemical (IHC) Staining

In brief, tissue microarray (TMA) sections were deparaffinized and immersed in 10 mM sodium citrate buffer (pH 6.0) in a microwave oven twice for 5 min to enhance antigen retrieval. After washing, slides were incubated with 0.3% H_2_O_2_ in methanol to quench the endogenous peroxidase activity. Slides were washed with phosphate-buffered saline (PBS) and incubated with anti-p-Akt (rabbit polyclonal antibody, Santa Cruz Biotechnology, Santa Cruz, CA, USA), anti-Snail (monoclonal mouse anti-Snail antibody, Biorbyt, Cambridge, UK), and appropriate negative control antibodies for 2 h at room temperature. After washing in PBS, slides were developed with a VECTASTAIN^®^ ABC (avidin-biotin complex) peroxidase kit (Vector Laboratories, Burlingame, CA, USA) and a 3,3,9-diaminobenzidine (DAB) peroxidase substrate kit (Vector Laboratories) according to the manufacturer’s instructions. All specimens were stained with H and E, which was used as a light counterstain.

### 4.4. Scoring of Immunoexpression

IHC results of p-Akt and Snail were classified into two groups according to the intensity and extent of staining. The intensity was scored semi-quantitatively as 0, negative; 1 point, weakly positive; 2 points, moderately positive; or 3 points, strongly positive. To determine the extent of Snail expression, 1000 consecutive malignant cells were counted in the area of the strongest staining. Numbers of cells with positive cytoplasmic staining for p-Akt and positive cytoplasmic and nuclear staining for Snail were recorded. The extent of p-Akt and Snail staining was semi-quantitatively scored as 0, positive in <1% of cells; 1 point, positive in 1%–25% of cells; 2 points, positive in 25%–50% of cells; 3 points, positive in 50%–75% of cells; or 4 points, positive in 75%–100% of cells. We then developed a p-Akt or Snail image score as previously described [6] by multiplying the intensity score (0–3 points) by the extent score (0–4 points) to represent the expression of p-Akt or Snail in cancer tissues. Low and high expression levels of p-Akt or Snail were respectively defined as 0–6 and 8–12 points. All sections were independently scored by the authors. 

### 4.5. Statistical Analysis

SPSS 17.0 statistical software (SPSS, Chicago, IL, USA) was used for all statistical analyses. Differences in the clinicopathological features and Akt image scores of the tumors were assessed using paired *t*-tests for continuous and categorical variables. A Cox proportional hazards regression model was used for a univariate analysis when assessing predictors of biochemical progression. The Kaplan-Meier method was used to compare the time to recurrence among the groups. The diagnostic value of potential biomarkers as predictors of biochemical failure was evaluated with receiver-operator characteristic (ROC) curves. The area under the ROC curve (AUC) was determined from the plot of sensitivity versus 1–specificity (true positive rate versus false positive rate) and is a measure of the predictability of a test. Statistical significance was defined at *p* < 0.05.

## 5. Conclusions

Our data demonstrate, for the first time, that p-Akt expression is highly correlated with Snail expression in a Taiwanese population with primary localized PCa. We also documented that p-Akt exerts its tumor-promoting role because of its associations with various aggressive clinicopathological characteristics and BCR in men with clinically-localized PCa. Our results highlight that, in patients with clinically-localized PCa, and a high p-Akt image score in cancer tissues, adjuvant radiotherapy or hormone therapy might be suggested to prevent early BCR. However, larger prospective cohorts and experimental studies are needed for comprehensive functional validation and better understanding of the clinical significance of p-Akt and Snail expression in PCa.

## Figures and Tables

**Table 1:** Characteristics of prostate cancer (PCa) patients at the pT2 stage who underwent a radical prostatectomy (RP).

Characteristic	Total (%)
Total number of patients	53
Median age at RP (years)	71, mean 70.7 ± 15.2 (48–88 y/o)
Mean preoperative PSA level (ng/mL)	10.31 (1–21.64 ng/mL)
Biochemical failure	25 (47.2)
Pathological stage	
T2a	6 (11.3)
T2b	4 (7.5)
T2c	43 (81.2)
Gleason score	
≥7	34 (64.2)
≤6	19 (35.8)
Snail image score	
≥8	35 (66)
≤6	18 (34)
Phosphorylated-Akt image score	
≥8	32 (60.4)
≤6	21 (39.1)
Median follow-up time (months)	99, mean: 71 ± 49.5 (53–184 m)

RP, radical prostatectomy; PSA, prostate specific antigen; y/o, years old; m, months.

**Table 2:** The association of phosphorylated (p)-Akt staining and clinicopathological features of prostate cancer (PCa) patients.

Characteristic	No. of Patients (%)		*p* Value
	pAkt Score ≥ 8	pAkt Score ≤ 6	
Total number of patients	32 (60.4)	21 (39.6)	
Age (years)			
<50	1	0	
50–59	5	3	
60–69	11	6	
≥70	15	12	
Pathological stage			
T2a	2 (6.25)	3 (14.3)	0.540
T2b	2 (6.25)	2 (9.5)	
T2c	28 (87.5)	16 (76.2)	
Gleason score			
≥7	28 (87.5)	8 (38.1)	0.024
≤6	4 (12.5)	13 (61.9)	
Snail image score			
≥8	29 (90.6)	6 (28.6)	0.035
≤6	3 (9.4)	15 (71.4)	
Recurrence	18 (56.3)	7 (33.3)	0.012
PSA, mean (ng/mL)	29.9	12.1	0.026

PSA: prostate specific antigen.

**Table 3:** Survival analyses of biochemical progression predictors in patients with prostate cancer at pT2 who underwent a radical prostatectomy (RP) according to a Cox proportional hazards regression model.

Factor	Hazard Ratio (95% CI)	*p* Value
PSA > 10 ng/mL	0.62 (0.12–3.19)	0.57
Pathological Gleason sum ≥ 7	1.18 (0.23–1.32)	0.05
Snail image score ≥ 8	1.31 (0.09–3.03)	0.06
P-Akt image score ≥ 8	3.12 (0.95–10.27)	0.05

## Abbreviations

BPH	Benign prostatic hyperplasia
BCR	Biochemical recurrence
EMT	Epithelial-mesenchymal transition
PCa	Prostate cancer
PI3K	Phosphatidylinositol 3′-kinase
PSA	Prostate-specific antigen
RP	Radical prostatectomy
TMA	Tissue microarrays
